# Trends in Suicides and Homicides in 21st Century America

**DOI:** 10.7759/cureus.61010

**Published:** 2024-05-24

**Authors:** Oluwasegun Akinyemi, Temitope Ogundare, Terhas Wedeslase, Brandon Hartmann, Eunice Odusanya, Mallory Williams, Kakra Hughes, Edward Cornwell III

**Affiliations:** 1 Surgery, Howard University Hospital, Washington, USA; 2 Psychiatry and Behavioral Sciences, Boston University School of Medicine, Boston, USA; 3 Surgery, Howard University College of Medicine, Washington, USA; 4 Medicine and Surgery, Howard University College of Medicine, Washington, USA

**Keywords:** united states of america, northeast united states, suicide mortality, suicide prediction, homicide, suicide prevention, suicide rates

## Abstract

Background: Violent deaths, including suicides and homicides, pose a significant public health challenge in the United States. Understanding the trends and identifying associated risk factors is crucial for targeted intervention strategies.

Aim: To examine the trends in suicides and homicides over the past two decades and identify demographic and contextual predictors using the Center for Disease Control and Prevention's Web-based Injury Statistics Query and Reporting System online database.

Methods: A retrospective analysis of mortality records from 2000 to 2020 was conducted, utilizing multivariate regression analyses. Covariates included age, race, sex, education, mental health conditions, and time period. Age-adjusted rates were employed to assess trends.

Results: Over the 20 years, there was an upward trajectory in suicide rates, increasing from approximately 10/100,000 to over 14/100,000 individuals, which is a notable increase among American Indians (100.8% increase) and individuals aged 25 years and younger (45.3% increase). Homicide rates, while relatively stable, exhibited a significant increase in 2019-2020, with African Americans consistently having the highest rates and a significant increase among American Indians (73.2% increase). In the multivariate regression analysis, Individuals with advanced education (OR= 1.74, 95% CI= 1.70 - 1.78), depression (OR = 13.47, 95% CI = 13.04 - 13.91), and bipolar disorder (OR = 2.65, 95% CI = 2.44 - 2.88) had higher odds of suicide. Risk factors for homicide include African Americans (OR = 4.15, 95% CI = 4.08 - 4.23), Latinx (OR = 2.31, 95% CI = 2.26 - 2.37), people aged 25 years and younger, and those with lower educational attainment.

Conclusion: This study highlights the changing demographic pattern in suicides and homicides in the United States and the need for targeted public health responses. Means restriction, universal suicide screening, addressing mental health stigma, and implementing broad interventions that modify societal attitudes toward suicide and homicides are essential components of a comprehensive strategy.

## Introduction

Violent deaths are a significant cause of death in the United States [1]. According to the Centers for Disease Control and Prevention's (CDC) Web-based Injury Statistics Query and Reporting System (WISQARS), there were about 72,000 violent deaths in the United States in 2020 [1]. A large proportion of these deaths were due to suicide and homicides. The estimated costs have been estimated to be about $104 billion annually [2]. In addition to the significant emotional and psychological impact on victims' families, suicide and homicide also affect the health and safety of whole communities [3].

Among persons 35-44 years, suicide was the fourth leading cause of death, among the three leading causes of death for people 10-34 years; homicide is the second leading cause of death for individuals aged 15-24 years and among the top four cause of death for people aged 1-14 years [1]. Several studies have shown that there are racial disparities in violent deaths in the United States, with Whites having the highest rates of suicide while African Americans have the highest rates of homicides [4-8]. However, in the past 10 years, there has been a rapid rise in suicide rates among ethnic majorities, especially American Indian or Alaskan Native and African Americans, compared to Whites [9]. Looking at trends in suicide and homicides, there was a decline in the homicide rate in the late 90s, but the rate has steadily been increasing since the 2000s; the suicide rate, on the other hand, has been steadily increasing [10,11].

The available data paints an alarming picture and calls for a concerted public health response to prevent violent deaths in the United States. Public health interventions depend on a solid understanding of the determinants and deterrents of a public health problem. In order to develop effective public health interventions to address violent deaths, especially suicide and homicide, we need a better understanding of the risk and protective factors [12-14]. There have been several studies that have reported the prevalence and distribution of suicide and homicide rates in the United States and shed light on racial disparities. However, few employ statistical modeling to identify risk factors for suicide and homicide in the general population.

This study aims to examine the data from the web-based Injury Statistics Query and Reporting System (WISQARS^TM^) over the past 20 years and to apply multivariate regression analyses to determine independent predictors of suicide and homicides in the United States. The goal is to generate results that are generalizable and will add to the evidence on the risk and protective factors of suicide and homicide in the United States, which will inform effective interventions and policies to address violent deaths.

## Materials and methods

A retrospective analysis of United States mortality records collated by the CDC from 2000 to 2020. We extracted all recorded instances of suicide and homicide as causes of death. These records provide a detailed account of each incident, including demographic information, cause of death, and other relevant details crucial for understanding the trends and factors associated with these deaths. We were interested in the past 20 years because of previous research that has reported a rising rate of suicide and homicide since the 2000s following an initial decline in these rates.

Outcome variable: suicide, defined as intentional infliction of bodily harm resulting in death, and homicide, defined as intentional harm inflicted by another person with a fatal outcome. 

Covariates: These included age, race, sex, level of education (categorized into high school, tertiary, which include college education, and advanced corresponding to postgraduate degrees), method of suicide or homicide, and presence of mental health conditions.

Data analysis: Data was analyzed using Stata version 16 (StataCorp LLC, College Station, TX) [15]. Descriptive statistics were described using frequency distribution tables and figures. We calculated age-adjusted suicide and homicide rates to account for changes in population growth over the 20 years (from 2000 to 2020). From 2015 to 2020, we conducted bivariate analyses for a subset of the data using Chi-square tests for categorical data. In addition, we conducted multivariate regression analyses to identify predictors of homicide and suicide, respectively. The model was adjusted for age, sex, gender, level of education, presence of mental health condition, and time period (pre-covid and post-covid). We report measures of association using odds ratio with 95% confidence intervals. For all tests, the level of significance was set at p<0.05.

Ethics statement: The study was conducted in accordance with the ethical standards of the institutional and/or national research committee and with the 1964 Helsinki Declaration and its later amendments or comparable ethical standards. Institutional Review Board approval was waived because the study was carried out on a national database that contained de-identified data and did not require informed consent or direct participation of patients.

## Results

Results

**Figure 1:**
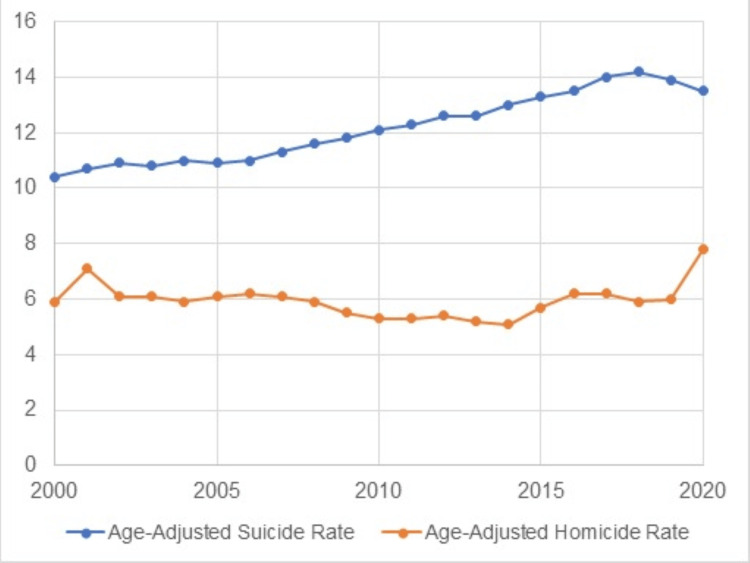
Trends in suicides and homicides in the United States from 2000 to 2020

The trend analysis (see Figure 1) of age-adjusted suicide and homicide rates shows contrasting trajectories. For the suicide rate, there was an upward trend, increasing steadily from approximately 10 per 100,000 individuals in 2000 to over 14 per 100,000 by 2020. In contrast, the homicide rate remained relatively stable, with slight fluctuations around six per 100,000 throughout the two decades. There was a slight increase in homicide rates observed around 2015, culminating in a sharper rise towards 2020. The trends suggest a concerning increase in suicides over the years, while homicides show potential signs of escalation towards the end of the period studied.

Table 1 shows the trend in suicide rates across age, gender, and ethnicity. Suicide rates in males have consistently been higher than females, 17.7/100,000 vs 4.0/100,000 in 2000 and 22.0/100,000 vs 5.5/100,000 in 2020. However, over the 20-year period, the suicide rate rose higher in females compared to males (37.5% vs 24.3%). Regarding race/ethnicity. Whites have a higher suicide rate compared to others; however, the rate of increase in African Americans is almost the same as that in Whites (37.5% vs 40%). The most significant increase in suicide rate was among American Indians, 100.8%. In 2000, persons 65 years and older had the highest rate of suicide, 14.6/100,000, while the suicide rate was similar among those aged 25-44 years and 45-64 years (13.4/100,000 vs 13.5/100,000). In 2020, individuals aged 25-44 years had the highest suicide rate (17.9/100,000), followed by those aged 45-64 years (17.6/100,000). However, those aged 25 years and younger had the most significant increase in the suicide rate over the 20-year period, with an increase of 45.3%. Adults 65 years and older had the lowest increase over the same period, 10.3%.

**Table 1:** Age-adjusted suicide rates (2000 to 2020) The observed trend in suicide rates across age, gender, and ethnicity.

Variable	Age-adjusted suicide rate (2000)	Age-adjusted suicide rate (2020)	% Increase (from 2000-2020)
Sex			
Males	17.7	22.0	24.3%
Females	4.0	5.5	37.5%
Race/Ethnicity			
White	12.0	16.8	40.0%
African American	5.6	7.7	37.5%
Latinx	5.9	7.5	27.1%
Asians-Pacific Islanders	5.6	6.8	21.4%
American-Indians	11.9	23.9	100.8%
Age			
< 25 Yrs	5.3	7.7	45.3%
25-44 Yrs	13.4	17.9	33.6%
45-64Yrs	13.5	17.6	30.4%
≥ 65Yrs	14.6	16.1	10.3%

Table 2 shows the trend in homicide across age, gender, and ethnicity. Homicide rate remained largely stable among females over the 20-year period (2.8/100,000 vs 2.9/100,000). In males, there was a 40% increase in homicide rate, from 9/100,000 in 2000 to 12.6/100,000. Whites recorded a 14.3% increase over the 20-year-period (2.8/100,000 vs. 3.2/100,000), while African Americans had a 45% increase (21.1/100,000 vs 30.6/100,000), American Indians had a 73.2% increase (8.2/100,000 vs 14.2/100,000). There was a reduction in homicide rate among Asian/Pacific Islanders (3.0/100,000 vs 1.7/100,000) and Latinx (7.5/100,000 vs 6.2/100,000). In 2000, individuals aged 25-44 years had the highest rate of homicide, 8.6/100,000, followed by those 25 years and younger, 6.6/100,000. This pattern remained the same in 2020, 9.8/100,000 and 8.0/100,000, respectively. However, individuals 45-64 years had the most significant increase in homicide rate, 35.0%, followed by those aged 25-44 years, 21.2%. Individuals aged 65 years and older had a reduction in the homicide rate over the 20-year period (-8.3%).

**Table 2:** Age-adjusted homicide rates (2000 to 2020)

Variable	Age-adjusted homicide rate (2000)	Age-adjusted homicide rate (2020)	% Change (from 2000-2020)
Sex			
Males	9	12.6	40.0%
Females	2.8	2.9	3.6%
Race/Ethnicity			
White	2.8	3.2	14.3%
African American	21.1	30.6	45.0%
Latinx	7.5	6.2	-17.3%
Asians-Pacific Islanders	3.0	1.7	-43.3%
American-Indians	8.2	14.2	73.2%
Age			
< 25 Yrs	6.6	8.0	21.2%
25-44 Yrs	8.6	9.8	14.0%
45-64Yrs	4.0	5.4	35.0%
≥ 65Yrs	2.4	2.2	-8.3%

In a sub-analysis of the data from 2015 to 2020 (see Table 3), comprising 11,618,554 deaths, suicide and homicide constituted 1.5% and 3.7%, respectively. Bivariate analyses showed a non-linear association between age and suicide (p<0.01) and age and homicide (p<0.01). Significant positive associations were also found between suicide/homicide and gender, race/ethnicity, educational attainment, depression, bipolar, anxiety, dementia, and the post-coronavirus disease 2019 (COVID-19) era. In the multivariate analyses (Table 4), compared to individuals 25 years and below, those aged 25-44 years had lower odds of suicide (OR = 0.84, 95% CI = 0.83 - 0.86), there were no significant associations for those aged 45-64 years and 65 years and older. Compared to Whites, African Americans (OR = 0.26, 95% CI = 0.25 - 0.27), Latinx (OR = 0.65, 95% CI = 0.64 - 0.67), Others (OR = 0.87, 95% CI = 0.84 - 0.89) had lower odds of suicide. Having tertiary (OR = 1.58, 95% CI = 1.56 - 1.60) or advanced education (OR = 1.74, 95% CI = 1.70 - 1.78) increased the odds of suicide. Individuals with depression (OR = 13.47, 95% CI = 13.04 - 13.91) and bipolar disorder (OR = 2.65, 95% CI = 2.44 - 2.88) have higher odds of suicide.

**Table 3:** Baseline characteristics of suicides and homicides in the United States (2015 to 2020) COVID-19- Coronavirus disease 2019; Data presented as N (%), bivariate analyses with p<0.05 as level of significance.

Variables	Total Deaths (N= 11,293,433)	Suicides (n= 211,027)	Homicides (n= 103,230)	Other causes (n=10,979,176)	p-value
Age					< 0.01
< 25	176,959 (1.5%)	29,885 (14.2%)	30,589 (29.6%)	116,485 (1.0%)	
25-44	425,220 (3.7%)	70,027 (33.2%)	47,839 (46.3%)	307,354 (2.7%)	
45-64	2,191,173 (18.9%)	70,630 (33.5%)	19,234 (18.6%)	2,101,309 (18.6%)	
≥65 yrs	88,814,338 (75.9%)	40,485 (19.2%)	5,568 (5.4%)	8,768,285 (77.6%)	
Female	5, 672, 812 (48.9%)	45,237 (21.4%)	20,156 (19.5%)	5,607,419 (49.7%)	<0.01
Race/ethnicity					< 0.01
White	6,757,636 (78.0%)	129,603 (79.7%)	22,010 (29.0%)	6,606,023 (78.4%)	
African American	1,099,893 (12.7%)	10,722 (6.6%)	38,659 (50.9%)	1,050,512 (12.5%)	
Latinx	551,158 (6.4%)	14,488 (8.9%)	12,563 (16.5%)	524,107 (6.2%)	
Others	230,699 (2.7%)	7,254 (4.5%)	2,511 (3.3%)	220,934 (2.6%)	
Unknown	26,449 (0.3%)	549 (0.3%)	257 (0.3%)	25,643 (0.3%)	
Education					<0.01
Pre-High School	7,232,258 (62.6%)	116,799 (55.6%)	80,177 (78.0%)	7,035,282 (62.6%)	
Tertiary	3,373,416 (29.2%)	77,003 (36.7%)	19,132 (18.6%)	3,277,281 (29.1%)	
Advanced	717,819 (6.2%)	12,572 (6.0%)	1,411 (1.4%)	703,836 (6.3%)	
Unknown	234,241 (2.0%)	3,754 (1.8%)	2,118 (2.1%)	228,369 (2.0%)	
Depression	74,670 (0.6%)	10,847 (5.1%)	29 (0.03%)	63,794 (0.6%)	<0.01
Bipolar	15,297 (0.1%)	1,283 (0.6%)	21 (0.02%)	13,993 (0.1%)	<0.01
Anxiety	30,931 (0.3%)	1,569 (0.7%)	34 (0.03%)	29,328 (0.3%)	<0.01
Dementia	774,833 (6.7%)	153 (0.1%)	65 (0.1%)	774,615 (6.9%)	<0.01
Obesity	235,713 (2.0%)	601 (0.3%)	247 (0.2%)	234,865 (2.1%)	<0.01
Post-COVID-19	5,826,445 (50.2%)	94,587 (44.8%)	53,154 (51.5%)	5,678,704 (50.3%)	< 0.01

**Table 4:** Factors associated with suicides and homicides in the United States (2015 to 2020) COVID-19: Coronavirus disease 2019; Multivariate logistic regression analyses, level of significance, p<0.05.

Variables	Suicides	Homicides
Age		
< 25 Yrs.	Reference	Reference
25-44 Yrs.	0.84 (0.83-0.86)	0.80 (0.78-0.82)
45-64 Yrs.	0.10 (0.10-0.11)	0.05 (0.050-0.052)
≥65 Yrs.	0.01 (0.013-0.014)	0.005 (0.004-0.005)
Female	0.341 (0.337-0.346)	0.34 (0.33-0.35)
Race/ethnicity		
White	Reference	Reference
African American	0.26 (0.25-0.27)	4.15 (4.08-4.23)
Latinx	0.65 (0.64-0.67)	2.31 (2.26-2.37)
Others	0.87 (0.84-0.89)	1.47 (1.41-1.54)
Education		
Pre-High School	Reference	Reference
Tertiary	1.58 (1.56-1.60)	0.76 (0.74-0.77)
Advanced	1.74 (1.70-1.78)	0.56 (0.52-0.59)
Depression	13.47 (13.04-13.91)	0.12 (0.08-0.19)
Bipolar	2.65 (2.44-2.88)	0.17 (0.10-0.29)
Anxiety	0.68 (0.62-0.73)	0.50 (0.34-0.74)
Dementia	0.04 (0.031-0.046)	0.13 (0.10-0.18)
Obesity	0.048 (0.044-0.053)	0.04 (0.03-0.04)
Post-COVID-19	0.82 (0.81-0.83)	1.02 (1.00-1.04)

For homicide, compared to individuals 25 years and younger, people aged 25-44 years (OR = 0.80, 95% CI = 0.78 - 0.82), there was no association for individuals ages 45-64 years and those 65 years and older. Females had lower odds of homicide (OR = 0.34, 95% CI = 0.33 - 0.35). Compared to Whites, African Americans (OR = 4.15, 95% CI = 4.08 - 4.23), Latinx (OR = 2.31, 95% CI = 2.26 - 2.37), Others (OR = 1.47, 95% CI = 1.41 - 1.54) have higher odds of homicides. There were reduced odds of homicide in individuals with tertiary education (OR = 0.76, 95% CI = 0.74 - 0.77) and advanced degrees (OR = 0.56, 95% CI = 0.52 - 0.59) compared to those with no high school diploma. There were 2% higher odds of homicide in the post-COVID-19 era (OR = 1.02, 95% CI = 1.00 - 1.04) compared to the pre-COVID-19 era.

## Discussion

The study aimed to describe the trends in suicide and homicide in the United States over a 20-year period. Our results show an upward trend in suicide rates in the past 20 years; the homicide rate largely declined before a sudden rise from 2019 to 2020. While Whites had the most significant suicide rate over the past 20 years, American Indians have the most significant increase in suicide rates. The rate of rise of suicide among African Americans was similar to that of Whites. Over the 20-year period, the age with the highest rate of suicide changed from individuals aged 65 years and older in 2000 to those aged 25-44 years, while the most significant increase was seen in people 25 years and younger. African Americans continued to have the most significant homicide rates over the 20-year period; however, homicide rates increased the most among American Indians. While individuals aged 25-44 years continue to record the highest rate of homicide, the homicide rate increased the most among those aged 45-64 years. In a sub-analysis of data, the highest risk for suicide was among males, people 25 years and younger, Whites, people with advanced education, and people with depression or bipolar disorder. The highest risk of homicide was among African Americans, males, people 25 years and younger, people with a high school diploma or lower, and post-COVID-19 era.

Our study is similar to other studies that have reported on the steady rise of suicide in the United States [10,16-18]. Studies have shown that the majority of completed suicides are firearm-related, accounting for one in two suicides in males and one in three among females [19-23]. Reducing the suicide rate, therefore, requires restriction of access to the most lethal means [24-26]. Laws and policies that reduce access to firearms are needed to reduce completed suicides [27-29]. Studies have shown that states with laws that restrict access to firearms have a lower rate of suicide compared to those that do not have such laws, with no increase in suicide from other methods [30-33]. A suicide attempt is often related to an acute stressor that involves unbearable psychological pain, and the individual wants to escape from it [34]. If individuals in such situations cannot access a lethal method to commit the act, they can often present for help or be accepting of help [20,35]. Public health approaches that modify risk factors not within an individual's volition are often the most successful [36]. Apart from laws and policies, universal screening for suicide is another effective public health prevention approach. Universal screening will aid the detection of at-risk individuals for whom targeted interventions can be implemented, and it helps to reduce disparities and bias by ensuring that all individuals are screened across all demographic groups, communities, and care settings [37-39]. Such target interventions include conversations with such individuals and their families and loved ones as appropriate to restrict access to firearms, including safety locks [40,41]. Other targeted interventions will involve safety planning, avoiding prescription of medications that are fatal in overdoses, and treatment of mental illness and/or substance use disorders [42,43]. Addressing the stigma of suicide and mental health is another broad public health intervention that can be effective in changing societal attitudes and norms about suicide [44-46].

This study has provided several insights into the changing demographics at risk for suicide. American Indians, African Americans, people aged 25 years and younger, and those between 25-44 years are at increased risk compared to twenty years prior. Several reasons may account for this demographic shift, including an increase in reporting or case identification, the increasing acceptance of mental health issues and reducing stigma, an increase in anxiety and depression, increasing economic challenges that disproportionately affect racial minorities, and the effect of climate change, social media, and its negative consequences on the mental health of adolescents and young adults [47-50]. These sociodemographic risk factors should help define at-risk populations for targeted public health interventions. Traditionally, suicide has been associated with lower education and low socioeconomic status [51,52]. However, in our study, those with higher educational attainment had the highest risk of suicide. This finding has been replicated in other studies [53-55]. Some explanation for this may be the mismatch between the expectations of individuals with advanced degrees in terms of economic remuneration and social class placement and their reality, which can lead to psychological distress and mental health issues [55-58]. Higher education comes at a cost in the United States, leading to massive student debts, which hinder most people from owning homes or even getting married [59]. College students often report loneliness, and people with college degrees marry later [60,61]. Social isolation and loneliness increase the risk of mental health conditions, including depression and anxiety, which increases the risk of suicide [62,63]. In addition, research shows that younger college graduates are more likely to be underemployed and to value their degree; this disappointment of not being able to achieve their goals may lead to increasing suicidal ideation and attempts [64]. Studies have shown associations between prospective economic conditions and suicide risk; if individuals with higher educational attainment do not expect better economic conditions in the future, they may have higher suicide risk [65]. Public health prevention approaches have consistently recognized the importance of health in all policies [66]. Educational and economic policies such as debt forgiveness and those that improve income may, therefore, have a profound impact on suicide prevention [28].

Regarding the risk of homicide, African Americans, younger individuals, and those with lower levels of education have the highest risk of homicide. These findings have been reported elsewhere, highlighting the importance of developing targeted interventions for these at-risk groups [67-69]. In thinking about effective interventions, it is crucial to consider intersectionality, which is how the presence of two or more demographic characteristics interact to increase the risk of homicide. African American males under the age of 35 years have the highest rate of homicide, and African Americans with lower educational attainment are at increased risk of homicide [67-69]. A racial justice lens is therefore needed in the design and implementation of effective homicide prevention strategies [70,71].

Compared to the previous years (2015 to 2019), 2020 was associated with a significant increase in homicide rates, similar to other studies [72,73]. COVID-19, which was declared a pandemic in early 2020, was associated with layoffs and economic hardship, especially for lower socioeconomic and racial/ethnic minorities, due to the lockdown [73-75]. There is a positive association between economic hardship and violence, including homicides [76]. This differential impact of COVID-19 lockdowns may account for the continuing disparities in homicide rates in the United States. Addressing structural racism and creating opportunities in the resource-poor neighborhood is necessary to address the upward trend in, and increased risk of, homicide and violence among racial/ethnic minorities [76,77]. As previously discussed earlier, applying epidemiologic principles of vector control to homicides cannot be overemphasized [78,79]. Firearms are the most prevalent vector for homicides, and restricting access to firearms is germane. In infectious disease epidemiology, we understand that transmitting pathogens to the host is an essential step for infectivity and an important target for disease control. We know that social determinants of health such as poverty, level of education, neighborhood-built environment, neighborhood poverty level, and education and economic policies are distal factors that predispose to violence [80]. While firearms are not the cause of homicide, access to firearms is a force multiplier [81]. If access to firearms were restricted, even when violence occurs, they are likely to be less fatal [82]. Applying effective strategies that have proven successful for other public health issues, such as tobacco smoking and motor vehicle accidents, should also be part of the public health strategy [83]. Firearm restriction is a controversial topic in the United States; however, we posit that data-driven public health messaging has the potential to change norms and population health behaviors [83]. Public health professionals need to engage stakeholders and communities using tailored messaging to influence health behaviors similar to strategies that have proven effective in tobacco control programs [84,85].

Studies have shown that climate change could increase violence [86,87]. The rising trend in violent deaths, suicide, and homicide reported in the study may be related to climate change [88,89]. There have been studies that report an increase in suicide and homicide in summer [28,90]. Over the 20-year period, temperature levels during the summer rose steadily, and this may influence suicide and homicide rates [91]. Exploring the impact of climate change on suicide and homicide is a future direction for suicide and homicide prevention studies [92]. Studies utilizing climate data over a long period may be able to parse out the effect of climate change on violent deaths.

Limitations

The study has several limitations that should be considered when interpreting the results from this study. First, the study was based on WISQARS^TM^ and is subject to underreporting and misclassifying the causes of death due to variations in reporting practices across states and over time [93 -95]. Second, due to the limitation of the database, our analysis may have excluded important variables such as access to mental health services, changes in law, and policies that directly or indirectly influence suicide and homicide rates. Third, sociodemographic risk factors may vary by suicide and homicide methods, and this was not accounted for in our study. Studies examining firearm-related suicide and homicide and other common methods of suicide and homicides are encouraged. Fourth, our analysis of the impact of COVID-19 on the homicide rate may underestimate or overestimate the risk due to the limitation of data. The effect of COVID-19 extended beyond 2020 and, therefore, was not captured in this study.

## Conclusions

This study reported on trends in homicide and suicide in the United States over a 20-year period and performed multivariate analyses to determine predictors of suicide and homicides. Suicide rates displayed an upward trend over the 20-year period, with Whites exhibiting the highest overall suicide rates. However, American Indians experienced the most significant increase in suicide rates. Suicide rates also surged among individuals aged 25 years and younger, marking a demographic shift from previous decades. The finding of a higher risk of suicide among people with advanced degrees challenges the traditional associations with suicide and may reflect changing risk factors for suicide. These findings emphasize the need for comprehensive and nuanced suicide prevention strategies that consider intersectionality. Means restriction, universal screening for suicide, addressing mental health stigma, and implementing broad interventions that modify societal attitudes toward suicide are essential components of a comprehensive strategy. Although stable for the most part, there was a sharp rise in homicide in 2020, with COVID-19 being a risk factor. There was also a substantial rise in homicide rates among American Indians. African Americans, people under 25 years, and people with lower educational attainment have the highest risk of homicides. These findings emphasize the need for a racial justice lens in tailoring interventions for high-risk groups to address these disparities.
